# Performance of multi-parametric magnetic resonance imaging through PIRADS scoring system in biopsy naïve patients with suspicious prostate cancer

**DOI:** 10.1080/2090598X.2022.2067615

**Published:** 2022-04-24

**Authors:** Amr Nowier, Hesham Mazhar, Rasha Salah, Mohamed Shabayek

**Affiliations:** a Department of Urology, Faculty of Medicine Ain-Shams University, Cairo, Egypt; b Department of Radiology, Faculty of Medicine, Ain-Shams University, Cairo, Egypt

**Keywords:** Prostate cancer, multi-parametric magnetic resonance imaging, PI-RADS, TRUS guided prostate biopsy

## Abstract

**Background:**

Use of multi-parametric magnetic resonance imaging (mp-MRI) and Prostate Imaging Reporting and Data System (PI-RADS) scoring system allowed more precise detection of prostate cancer (PCa). Our study aimed at evaluating the diagnostic performance of mp-MRI in detection of PCa.

**Methods:**

Eighty-six patients suspected to have prostate cancer were enrolled. All patients underwent mp-MRI followed by systematic and targeted trans-rectal ultrasound (TRUS) guided prostate biopsies. Sensitivity, specificity, positive predictive value (PPV), negative predictive value (NPV) and accuracy of mp-MRI were evaluated.

**Results:**

Forty-six patients (53.5%) had prostate cancer on targeted and systematic TRUS biopsies. On mp-MRI, 96.6% of lesions with PI-RADS < 3 revealed to be benign by TRUS biopsy, 73.3% of lesions with PI-RADS 4 showed ISUP grades ≥1, whereas all PI-RADS 5 lesions showed high ISUP grades ≥ 3. For PI-RADS 3 lesions, 62.5% of them revealed to be benign and 37.5% showed ISUP grades ≥1 by TRUS biopsy. PI-RADS scores ˃3 had 69.57% sensitivity and 85% specificity for detection of PCa. On adding the equivocal PI-RADS 3 lesions, PI-RADS scores ≥3 had higher sensitivity (97.83%), but at the cost of lower specificity (32.5%).

**Conclusion:**

Mp-MRI using PI-RADS V2 scoring system categories ≤3 and >3 could help in detection of PCa. PI-RADS 3 lesions are equivocal. Including PI-RADS lesions ≥3 demonstrated higher sensitivity, but at the cost of lower specificity for mp-MRI in diagnosis for Pca.

**Abbreviations:**

CDR: cancer detection rates; DRE: digital rectal examination; ISUP: international society of urological pathology; mp-MRI: multi-parametric magnetic resonance imaging; NPV: negative predictive value; PCa: prosatate cancer; PI-RADS: Prostate Imaging Reporting and Data System; PPV: Positive predictive value; PSA: prostate specific antigen; TRUS: transrectal ultrasound.

## Introduction

Prostate cancer (PCa) is the most common cancer among men worldwide [1]. Systematic 10 to 12 cores prostate biopsy is the standard method for diagnosis of PCa [2]. The 10 to 12 core prostate biopsy demonstrated cancer detection rates (CDR) of 31–42%, but still has the risk of a false-negative prostate biopsy [3,4]. Many studies were performed to improve the CDR by increasing the number of cores, but this strategy proved to be ineffective as it identified more insignificant tumors [5].

Multi-parametric magnetic resonance imaging (mp-MRI) of the prostate has been studied as an alternative to increase CDR even in patients with a previously negative prostate biopsy [6]. Different aspects of mp-MRI are evaluated with the following characteristics [7]:

- Diffusion-weighted imaging (DWI): important to determine the peripheral zone (PZ) lesions.
- T2 weighted image (T2WI): important to determine transitional zone (TZ) lesions.
- Dynamic contrast-enhanced (DCE): ineffective in assessment of TZ and low-volume lesions [8].
- Magnetic resonance spectroscopy (MRS): time consuming and expensive [9].

Previously, different scores for mp-MRI were used to categorize the level of suspicion of the presence of PCa [10,11]. European Society of Urogenital Radiology (ESUR) published guidelines based on expert consensus in 2012, to standardize a score for evaluation and reporting of prostate MRI, known as the Prostate Imaging Reporting and Data System (PI-RADS) [12]. Since then, many clinical and research programs have validated this score. In 2015, the PI-RADS Steering Committee developed an updated version (PI-RADS V2) to overcome some of the limitations of PI-RADS V1 [13]. Purpose of our study was to evaluate the diagnostic performance of PIRADS V2 scores in patients with suspicious PCa.

## Materials and methods

### Study subjects

Retrospective analysis of prospectively collected data of 86 patients with suspicious prostate cancer recruited from the Urology department, Faculty of Medicine, Ain Shams University between August 2019, and December 2020 was evaluated. All participants were Egyptians. All patients were suspected to have prostate cancer based on elevated prostate specific antigen (PSA) and/or positive digital rectal examination (DRE). Patients with metallic prosthesis, pacemaker, or abnormal kidney function or previous negative biopsies were excluded. Our study was carried out after local ethical committee approval (FMASU M S 65/2019) and obtaining informed consent from all patients included in our study.

### Methods

All patients were subjected to full history taking, careful digital rectal examination, basic laboratory investigations, serum PSA, mp-MRI. PI-RADS v.2 scores were reported by senior radiologist for all patients in 8 sites of the prostate; right base, right mid zone, right apex, left base, left mid zone, left apex, right and left anterior fibromuscular stroma. This was followed by systematic and targeted transrectal ultrasound (TRUS) guided prostate biopsy using the cognitive fusion technique.

#### Mp-MRI protocol

The study was performed on a 3.0-T MRI system (MAGNETOM Skyra; Siemens Healthcare, Erlangen, Germany) with an 18-element body phased array coil and a 32-element spine array coil. Before contrast injection, anatomical MRI was performed including sagittal and axial T2-weighted (T2W) HASTE (half-Fourier acquisition single shot turbo spin-echo) with controlled respiration, without fat-suppression (FS); coronal T2-weighted HASTE without FS; axial 3D T1-twist dynamic study in free breath for about 50 frames in 3–4 minutes. Gadolinium-based contrast was given intravenously by means of a power injector (Ulrich Medical® Tennessee TM, Germany) at an infusion rate of 1 ml/s. Then, pre-contrast T1- mapping with two flip angles were obtained. Subtracted images were computed if needed.

#### TRUS-guided prostate biopsy

Systematic and targeted TRUS-guided prostate biopsies using the cognitive fusion technique were performed by an experienced senior urologist with more than 5 years experience. All patients had 12 cores systematic TRUS-guided prostate biopsy in addition to 2–3 targeted biopsies from PIRADS ≥3 on mp-MRI. Biopsy specimens were then assessed by two different expert pathologists in urologic oncology, who were blinded to MRI examination results, before reporting the International Society of Urological Pathology (ISUP) 2014 updated Gleason score grading system [14,15].

### Statistical analysis

The collected data was revised, coded, tabulated, and evaluated using Statistical Package for Social Science (SPSS 25). Mean, Standard deviation (± SD) and range were reported for parametric numerical data, whereas frequency and percentage of non-numerical data. Sensitivity, specificity, positive predictive value (PPV) and negative predictive value (NPV) of mp-MRI were evaluated. Kappa statistics was used to evaluate the agreement between two investigational methods. Kappa’s value 0.4–0.75 meant fair to good agreement, whereas Kappa’s value below 0.4 meant poor agreement.

## Results

Our current study included 86 patients with suspicious PCa. The basic characteristics of the patients included were described in (Table 1). Mp-MRI revealed that 72 patients (83.7%) had one or more PI-RADS lesions ≥3, whereas 14 patients had PIRADS score <3 in all reported sites. After targeted and systematic biopsies, only 46 patients (53.5%) were proved to have PCa.

**Table 1.** Basic Characteristics of studied population.

		n = 86
Age	Mean ±SD	63.07 ± 7.28
	Range	49–79
DRE	NAD	40 (46.5%)
	Firm	33 (38.4%)
	Hard	13 (15.1%)
PSA	Mean ±SD	10.07 ± 4.62
	Range	3.5–34
PSA density	Mean ±SD	0.19 ± 0.09
	Range	0.05–0.69
Prostate volume	Mean ±SD	55.37 ± 16.51
	Range	26–105

**SD**: standard deviation, **DRE**: digital rectal examination, **NAD**: no abnormality detected, **PSA**: prostate specific antigen.

688 sites were evaluated in mp-MRI of 86 patients and PIRADS V.2 scores were reported. 591 (85.9%) sites were proved to be benign by TRUS biopsies, whereas 97 (14.1%) sites harbored PCa. 134 PI-RADS ≥3 lesions were detected by mp-MRI, of which 64 lesions were classified as PI-RADS 3, whereas 60 lesions were classified as PI-RADS 4, and only 10 lesions were classified as PI-RADS 5.

In lesions with PI-RADS score <3, there was a very low likelihood of presence of any PCa (3.4%). However, in patients with PIRADS score 3, 4 and 5, PCa was detected in 37.5%, 73.3% and 100% respectively. We also found statistically significant correlation between PIRADS score and ISUP grade (P < 0.001). (Table 2)

**Table 2.** Correlation between PIRADS score & ISUP grade results in all studied lesions.

Mp-MRI (PIRADS score)	< 3	3	4	5	Agreement	
ISUP grade(GS)	n (%)	n (%)	n (%)	n (%)	Kappa	P value
Benign	535 (96.6)	40(62.5)	16(26.7)	0	0.345	<0.001
1 (3 + 3)	7 (1.3)	12 (18.8)	9 (15)	0		
2 (3 + 4)	9 (1.6)	8 (12.5)	17(28.3)	0		
3 (3 + 5, 4 + 4, 5 + 3)	2 (0.4)	2(3.1)	4(6.7)	1 (10)		
4 (4 + 5, 5 + 4)	1 (0.2)	2 (3.1)	14(23.3)	9 (90)		
5 (5 + 5)	0	0	0	0		
Total (n = 688)	554 (80.5%)	64 (9.3%)	60 (8.7%)	10 (1.5%)		

**TRUS**: transrectal ultrasound, **ISUP**: international society of urological pathology, **mp-MRI**: multiparametric magnetic resonance imaging, **PIRADS**: prostate imaging-reporting and data system score, **GS**:Gleason score

**Figure 1.**
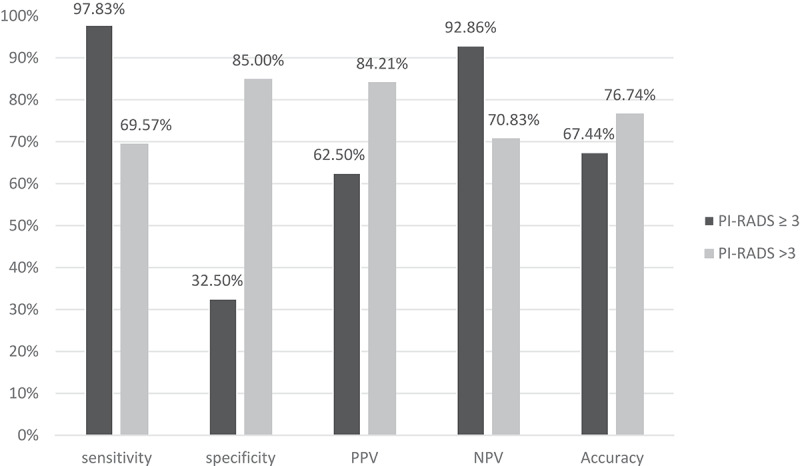
Diagnostic performance of PIRADS score ˃ 3 versus PIRADS score ≥3 on mp-MRI and detection of prostate cancer.

We found good agreement between PI-RADS score ˃3 on mp-MRI and detection of prostate cancer (kappa = 0.539) and mp-MRI achieved sensitivity of 69.57%, specificity of 85%, PPV of 84.21%, NPV of 70.83% and accuracy reached 76.74% in detection of prostate cancer compared to TRUS biopsy (Table 3, Figure 1). It is worth mentioning that adding the equivocal PI-RADS score of 3, performance of mp-MRI with PI-RADS score of ≥3 demonstrated higher sensitivity (97.83%), but lower specificity (32.50%). PPV and NPV were 62.50% and 92.86% respectively and accuracy reached 67.44% (Table 4, Figure 1).

**Table 3.** Correlation between PI-RADS score >3 lesions on mp-MRI and results of TRUS Biopsy.

PI-RADS	TRUS Bx		Total	Agreement	
	Negative	Positive		Kappa	p value
Negative (≤3)	34 (85%)	14 (30.43%)	48 (55.81%)	0.539	<0.001
Positive (>3)	6 (15%)	32 (69.57%)	38 (44.19%)		
Total	40 (100%)	46 (100%)	86 (100%)		

**TRUS**: transrectal ultrasound, **mp-MRI**: multiparametric magnetic resonance imaging, **PIRADS**: prostate imaging-reporting and data system score.

**Table 4.** Correlation between PI-RADS ≥3 on mp-MRI and results of TRUS Biopsy.

PI-RADS		TRUS Bx		Total	Agreement	
		Negative	Positive		Kappa	p value
	Negative (<3)	13 (32.5%)	1 (2.17%)	14 (16.28%)	0.317	<0.001
	Positive (≥3)	27 (67.5%)	45 (97.83%)	72 (83.72%)		
Total		40 (100%)	46 (100%)	86 (100%)		

**TRUS**: transrectal ultrasound, **mp-MRI**: multiparametric magnetic resonance imaging, **PIRADS**: prostate imaging-reporting and data system score.

## Discussion

PCa is the most diagnosed cancer in men [1]. However, the available diagnostic modalities have unfavorable sensitivities and specificities. PSA has low specificity of 36%, so elevated PSA does not necessarily mean the presence of a malignant lesion, and TRUS-guided biopsy may underestimate extent and grade of prostate cancer [12]. Recently, mp-MRI has been widely used for the diagnosis of PCa and demonstrated high sensitivity in detection of PCa [1]. Adding a standardized reporting method through PI-RADS scoring system to mp-MRI increased its ability to detect PCa [16].

In our study mp-MRI done for all cases with suspicious prostate cancer before TRUS guided biopsy to evaluate the diagnostic performance of mp-MRI (PI-RADS ≥3 score) to detect PCa using TRUS-guided biopsy as a reference standard. Our study demonstrated statistically significant correlation between PI-RADS score and ISUP grade, where lesions with PI-RADS ˃3 demonstrated high percentage of cancer by TRUS biopsy (73.3% and 100% respectively). Most of lesions with PI-RADS < 3 were benign by TRUS biopsy (96.6%) and this may help to avoid unnecessary TRUS guided biopsied in these patients. These results were in concordance with the study of Junker and colleagues who showed that 92% and 100% of lesions with PI-RADS score ˃ 3 were found as high grade PCa by TRUS biopsy and 97% of lesions with PI-RADS score < 3 were benign by biopsy [17].

Regarding the equivocal PI-RADS 3 lesions; our study included 34 patients with 64 PI-RADS 3 lesions, 62.5% of them revealed to be benign and 37.5% showed ISUP grades ≥1 by TRUS biopsy. This also agreed with Aslam and colleagues who demonstrated that 56% of patients with PI-RADS 3 had non-malignant findings and 43% of patients had malignant prostatic adenocarcinoma on transrectal/transperineal ultrasound-guided biopsy [18].

PI-RADS scores ˃3 demonstrated acceptable ability to detect PCa compared to TRUS biopsy with good sensitivity (69.57%), specificity (85%), PPV (84.21%), and NPV (70.83%) and accuracy reaching 76.74%. However, including lesions with PI-RADS score ≥3 for detection of PCa raised sensitivity of mp-MRI to 97.83%, but at the cost of lower specificity of 32.5%. Patel and colleagues demonstrated that including lesions with PI-RADS ≥3 had high sensitivity of 81.25%, but low specificity of 32.26%. They also reported that PI-RADS ˃ 3 lesions were associated with low sensitivity (43.7% and 37.5% respectively), but higher specificity (64.5% and 100% respectively) [19]. This was also confirmed by Youn and colleagues who demonstrated increased sensitivity but decreased specificity with the use of PI-RADS score of ≥3 compared to PI-RADS score of ˃ 3. They reported that sensitivity, specificity, PPV, NPV, and accuracy for PI-RADS score of ≥3 were 92.3%, 58%, 62.3%, 90.9% and 72.7% respectively compared to 84.6%, 81.2%, 77.2%, 87.5% and 82.6% respectively for PI-RADS score of ˃ 3 [20].

Main limitation of our study was the small population, so large multicentric studies are recommended to ascertain our results. Another limitation was lack of comparing results of TRUS biopsy to final histological findings of radical prostatectomy specimen.

## Conclusion

Mp-MRI using PI-RADS V2 scoring system categories ≤3 and >3 could help in diagnosis of PCa. We can safely refrain patients with PI-RADS lesions ˂3 lesions from prostate biopsies. PIRADS 3 lesions are equivocal. Evaluating PIRADS lesions ˃3, achieves high specificity for dianosis of Pca. However including PI-RADS lesions ≥3 demonstrated higher sensitivity, but at the cost of lower specificity for mp-MRI in diagnosis of PCa.
